# Assessing the Applicability of Lanthanide-Based Upconverting Nanoparticles for Optically Monitoring Cement Hydration and Tagging Building Materials

**DOI:** 10.1021/acsomega.5c02236

**Published:** 2025-07-16

**Authors:** Philipp Kossatz, Alexander Mezhov, Elina Andresen, Carsten Prinz, Wolfram Schmidt, Ute Resch-Genger

**Affiliations:** † Division Biophotonics, Federal Institute for Materials Research and Testing (BAM), Richard-Willstaetter-Str. 11, D-12489 Berlin, Germany; ‡ Division Technology of Construction Materials, Federal Institute for Materials Research and Testing (BAM), Unter den Eichen 87, D-12205 Berlin, Germany; § Division Structure Analysis, Federal Institute for Materials Research and Testing (BAM), Richard-Willstaetter-Str. 11, D-12489 Berlin, Germany; ∥ Institute for Chemistry and Biochemistry, Free University Berlin, Arnimallee 20, D-14195 Berlin, Germany

## Abstract

Chemically stable, lanthanide-based photon upconversion micro- and nanoparticles (UCNPs) with their characteristic multicolor emission bands in the ultraviolet (UV), visible (vis), near-infrared (NIR), and short-wave infrared (SWIR) are promising optical reporters and barcoding tags. To assess the applicability of UCNPs for the monitoring of early stage cement hydration processes and as authentication tags for cementitious materials, we screened the evolution of the luminescence of self-made core-only NaYF_4_:Yb,Er UCNPs and commercial μm-sized Y_2_O_2_S:Yb,Er particles during the first stages of cement hydration, which largely determines the future properties of the hardened material. Parameters explored from the UCNP side included particle size, morphology, surface chemistry or coating, luminescence properties, and concentration in different cement mixtures. From the cement side, the influence of the mineral composition of the cement matrix was representatively examined for ordinary Portland cement (OPC) and its constituents tricalcium aluminate (C3A), tricalcium silicate (C3S), and gypsum at different water to cement ratios. Based on reflection and luminescence measurements, enabling online monitoring, which were complemented by XRD and isothermal heat-flow calorimetric measurements to determine whether the incorporation of these particles could impair cement hydration processes, well suited lanthanide particle reporters could be identified as well as application conditions. In addition, thereby the reporter influence on cement hydration kinetics could be minimized while still preserving a high level of information content. The best performance for the luminescence probing of changes during early stage cement hydration processes was observed for 25 nm-sized oleate (OA)-coated UCNPs added in a concentration of 0.1 wt %. Higher UCNP amounts of 1.0 wt % delayed cement hydration processes size- and surface coating-specifically in the first 24 h. Subsequent luminescence stability screening studies performed over a period of about one year support the applicability of UCNPs as optical authentication tags for construction materials.

## Graphical abstract

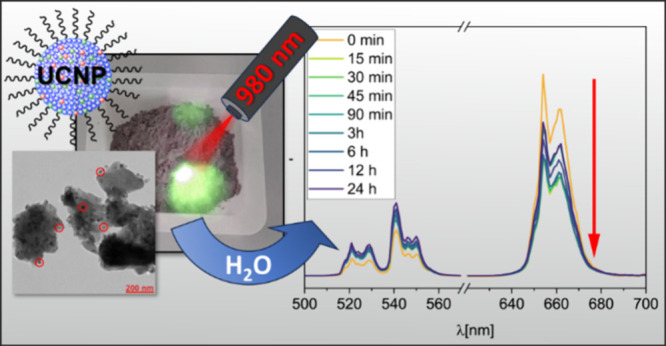

## Introduction

Spectrally shifting lanthanide-doped photon upconversion nanoparticles (UCNPs) have emerged as a promising class of novel optical probes and reporters in the life and material sciences^1,2^ and barcodes due to their characteristic multitude of emission bands in the ultraviolet (UV), visible (vis), near-infrared (NIR), and short-wave infrared (SWIR) wavelength region.^3,4^ UCNPs, which commonly consist of an inert, transparent, low phonon energy host matrix such as NaYF_4_, codoped with absorbing and emissive lanthanide ions such as Yb^3+^ and Er^3+^ or Tm^3+^ acting as sensitizers and activators,^5^ reveal a nonlinear upconversion luminescence (UCL) shifted to shorter wavelengths in response to multiphoton excitation in the NIR, e.g., at 980 nm. In addition, these materials exhibit a conventional linear down-shifted luminescence (DSL) at longer wavelengths than the excitation light upon direct excitation of the emissive lanthanides.^6^ The UCNP emission pattern and color can be fine-tuned by particle size and shape, crystal phase of the host, chemical composition, i.e., dopant ion nature and concentration, and particle architecture^7^ as well as by excitation power density.^8^ As the presence of luminescence quenchers such as water^9,10^ or parameters like temperature^11^ and pressure^12^ can induce changes in the UCNP emission profiles and luminescence lifetimes, these nanocrystals can also be exploited for environmental sensing. These properties, together with other advantageous features such as a high chemical and photochemical stability and the absence of blinking, have meanwhile initiated the development of a wide variety of UCNP-based optical sensors. In addition, due to their unique optical properties, UCNPs can be applied as optical barcodes, e.g., for security inks and printable anticounterfeiting and security tags,^13,14^ and have been suggested for the encoding of certain types of microplastics for small scale recycling applications.^15^

Among the worldwide most important and most frequently used inorganic material systems are mass building materials such as concrete and their constituents like cement or other mineral binders, which are produced and used on a scale of over 4 billion tons each year.^16^ Despite the widespread use of cement and many standards regulating its quality, there is still a lack of simple methods for *in situ* online monitoring of the complex, dynamic processes involved in cement hydration. Also, for such building materials, tags to encode their source, to ensure their quality, and to track the cement life cycle become increasingly relevant.^17^ This calls for monitoring techniques such as relatively simple, inexpensive, fast, sensitive, and noninvasive optical methods like fluorescence.^18,19^ Fluorescence methods commonly rely on a molecular or nanoscale reporter for signal generation, which exhibits absorption and/or fluorescence properties which respond to changes in the luminophore environment or the presence of a target.^1,2^ Although being straightforward and suitable for online *in situ* measurements, fluorescence monitoring can present a considerable challenge for cementitious materials due to the complex and varying physicochemical environment during cement hydration and the harsh conditions with very high pH values exceeding 13,^20^ exothermic reactions, temperatures up to 80 °C, high ionic strength in the aqueous phase of the binder paste, and significant shear forces and changes during transportation and casting. However, we could recently demonstrate that optical spectroscopy in conjunction with fluorescent organic dyes such as 2′,7′-difluorofluorescein or a cyanine dye as fluorescent reporters and probes can be utilized to monitor hydration-induced processes and changes in the early phase of cement formation.^21,22^ Although the use of optical reporters for studying cementitious systems is not yet widely established, there are also reports from other research groups on employing analyte-responsive organic dyes such as sensor dyes for pH or chloride anions for monitoring processes in cementitious materials including cement corrosion.^23–25^ Also fluorescent carbon quantum dots have been used in cement matrices, thereby focusing on modifying the structural properties of cement and not acting as optical reporters.^26^ In addition, a first concept for authentication tags and tracers for cementitious starting materials using polymer microparticles stained with different organic dyes has been presented, suggesting the use of flow cytometry for tracer detection.^19^

The results of our first fluorescence studies with organic fluorophores and the increasing interest in optical reporters and tags for building materials encouraged us to explore the potential of chemically very stable lanthanide-based particles of different sizes and surface chemistry such as self-made NaYF_4_:Yb,Er UCNPs and commercial Y_2_O_2_S:Yb,Er microparticles as optical probes for hydration processes in cementitious materials. Thereby, we also aimed for exploiting their favorable optical properties emphasized in the first section such as their unrivaled multicolor emission in the UV/vis/NIR/SWIR, consisting of characteristic, sharp emission bands with an environment-sensitive luminescence spectral and intensity distribution, together with their NIR excitability and high chemical robustness.^27^ As a typical cementitious system, we chose ordinary Portland cement (OPC) with its constituents tricalcium aluminate (C3A), tricalcium silicate (C3S), and gypsum at different water to cement ratios. Special emphasis was dedicated to the first hours of cement hydration as the most important period in which the future properties of hardened material are being formulated. Particle reporter stability was optically determined after the mixing with the cementitious materials over the course of cement hydration and then monitored over a period of about one year. In addition to optical methods, suitable for online *in situ* monitoring, we employed XRD (powder X-ray diffractometry) and isothermal heat-flow calorimetric measurements to determine whether the incorporation of UCNPs could affect cement hydration processes. Even though the complex nature of the cementitious environment makes a direct correlation between emission changes of UCNPs and individual chemical processes during cement hydration challenging, our results indicate the considerable potential of UCNPs for the luminescence probing of changes during these early stage cement hydration processes. At the same time, the importance of UCNP size and surface coating for optimum performance and the long-term applicability of such lanthanide nanoparticles as encoding and authentication tags for construction materials was revealed.

## Materials and Methods

### Materials

YCl_3_·6H_2_O (99.99%), YbCl_3_·6H_2_O (99.99%), ErCl_3_·6H_2_O (99.99%), oleic acid (90% technical grade), NaOH (98%), and HCl (37%, technical grade) were purchased from Sigma-Aldrich. 1-Octadecene (ODE, 90% technical grade) and NH_4_F (99.99%) were obtained from Alfa Aesar. Chloroform, acetone, and ethanol were purchased from Carl Roth GmbH. Y_2_O_2_S:Yb,Er microparticles were purchased from Leuchtstoffwerke Breitungen (product LP-UC23G-10-00). Ordinary Portland cement (OPC, CEM I 42.5 R), with a specific gravity of 3.1 g/cm^3^, a median particle size (d50) of 15.33 μm, and a Blaine value of 3,300 cm^2^/g, was obtained from CEMEX Zement GmbH, Rüdersdorf. The chemical composition of the OPC used is listed in Table 1.

**Table 1:** Chemical Composition of Cement in Weight Percent (wt %) According to DIN-EN 196

component	CaO	SiO_2_	Al_2_O_3_	Fe_2_O_3_	MgO	TiO_2_	K_2_O	Na_2_O	P_2_O_5_	Mn_2_O_3_	SO_3_	IR	LOI
Content (wt %)	62.57	19.11	4.28	2.55	1.95	0.24	1.01	0.29	0.26	0.05	3.28	0.81	3.58

### Synthesis of NaYF_4_:Yb^3+^,Er^3+^ UCNPs

The synthesis of the UCNPs was performed according to a modified procedure from Wilhelm et al.^28^ The salts YCl_3_·6H_2_O (3.9 mmol), YbCl_3_·6H_2_O (1.0 mmol), and ErCl_3_·6H_2_O (0.1 mmol) were dissolved in ∼10 mL of methanol by sonication. This solution was transferred into a 250 mL three-necked flask, mixed with oleic acid (OA; 30 mL of OA for 25 nm UCNP and 20 mL of OA for 55 nm UCNPs) and 80 mL of 1-octadecene (ODE) and heated to 160 °C under a vacuum. A homogeneous, clear solution was formed after 30 min at 160 °C under a vacuum. The reaction mixture was then cooled to room temperature, and 10 mL of methanol containing NaOH (0.25 M) and NH_4_F (0.4 M) was added. The resulting colloidal suspension was stirred for 30 min at 120 °C under a gentle argon flow and then heated to reflux at 325 °C for 1 h. After cooling to room temperature, the formed hexagonal oleate-capped UCNPs were precipitated by addition of ethanol and isolated via centrifugation at a relative centrifugal force (RCF) of 1000*g* for 10 min. The oleate-capped UCNPs were purified by three cycles of precipitate with 10 mL of chloroform and reprecipitation by the addition of 40 mL of ethanol. The product was dried for 24 h under air. Removal of the OA surface ligands was performed according to a modified literature procedure by adding 1 M HCl to a suspension of 1 g UCNP in 50 mL of H_2_O until a pH value of 4 was reached, followed by stirring for 4 h.^10^ The suspension was extracted three times with 30 mL of Et_2_O before the UCNPs were removed by centrifugation. The product was dried for 24 h under air.

### Preparation of UCNP-Cement Powders

Cement powders with a UCNP content of 1 wt % were prepared from all five particle samples by grinding 4.96 g of cement and 40 mg of dried UCNPs with pestle and mortar for 3 min. UCNP-cement powders containing 0.1 wt % of UCNPs were prepared in the same way by combining 4.996 g of cement and 4 mg of UCNPs.

### Structure-Analytical and Optical-Spectroscopic Characterization of the UCNPs

Transmission electron microscopy (TEM) images of the UCNPs and the UCNP-cement powder samples were obtained with a Talos F200S Microscope (Thermo Fisher Scientific) using an acceleration voltage of the electron beam of 200 kV. The samples were prepared by dropping UCNP dispersions (*c* = 1 mg/mL in water) onto a 3 mm copper grid (lacey, 400 mesh) and allowing them to dry under air at RT. The TEM images were analyzed with the software ImageJ. The average particle size of the OA-capped UCNPs was determined from EM micrographs. Thereby, the particle area was automatically measured using a fixed threshold based on unprocessed image intensity histograms and size distribution descriptors (e.g., Fere*t*_max_ and Fere*t*_min_). The obtained diameters were then plotted in the form of a histogram, which was subsequently fitted with a Gaussian curve. The mean (μ) and standard deviation (σ_*x*_) of this curve were taken as a representative particle size for the respective sample.

### Optical Spectroscopy of UCNPs and UCNP-Cement Samples

Steady state luminescence measurements of the UCNPs dispersed in cyclohexane (OA-capped UCNPs) or in water (ligand-free UCNPs) were performed with a calibrated fluorometer FLS980-xD2-stm (Edinburgh Instruments), utilizing an excitation wavelength of 980 nm provided by an 8 W 978 nm laser diode operated in the CW (continuous wave) mode equipped with a red-sensitive photomultiplier tube (PMT; Model H10720-20). For the luminescence measurements of the UCNP-cement samples, a custom-built miniaturized spectroscopic setup schematically represented in Figure 1 was employed, thereby utilizing the spectrofluorometer FLS980-xD2-stm as an excitation light source and detection system. To prevent heating of the samples due to light absorption by water molecules at 980 nm and enable a comparison of the measured fluorescence intensities, all measurements of the UCL of Er^3+^ and the down-shifted luminescence (DSL) of Yb^3+^ (Yb-DCL) of the UCNP-cement samples were performed at the same low excitation power density (*P*) of 7.07 W/cm^2^ at 980 nm, which was also employed for the measurement of the UCL spectra of the UCNP dispersions. For the optical monitoring of early phase cement hydration, 22 mg of UCNP-containing cement powder was placed on a microscope slide (26 × 76 mm, with indentation of 1.20–1.50 mm) followed by addition of 11 μL of deionized water. The pastes were mixed for 1 min, before the mixtures were covered with a coverslip (20 × 20 mm), which was then fixed with an epoxy resin to prevent the evaporation of water or the carbonation of the pastes. The luminescence emission spectra were acquired *in situ* 3 min after the start of cement hydration. For the evaluation of the luminescence data, the strongest green and red emission bands of Er^3+^ at 525, 540, and 654 nm were exploited, i.e., integrated and plotted as a function of hydration time.

**Figure 1:**
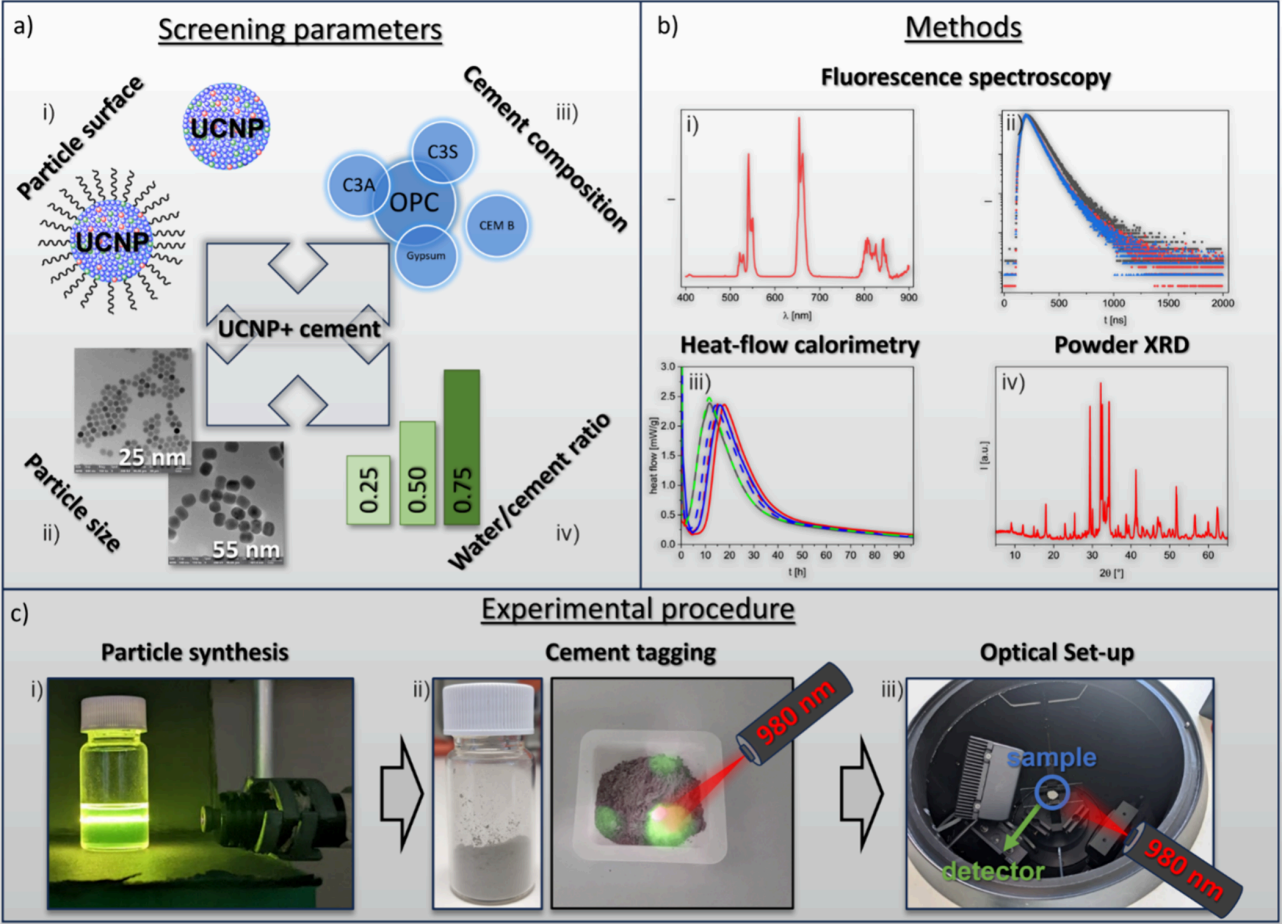
Overview of the screening studies utilized for identifying the best suited UCNPs and UCNP-cement systems for cement hydration probing and measurement conditions. **a)** Screening parameters explored from the particle and cement side: (i) particle surface coating (here oleate-capped and ligand free particles), (ii) particle size, (iii) cement composition including pure cement constituents, (iv) water-to-cement ratio, and UCNP concentration. **b)** Analytical methods employed to evaluate the particle behavior in cement: (i) steady-state and (ii) time-resolved fluorescence spectroscopy, (iii) heat-flow calorimetry, and (iv) powder XRD. The latter two methods were utilized to assess a possible influence of the particles on cement hydration processes; **c)** Overview of the luminescence measurements: Images from the core experimental steps in the procedure showing (i) freshly synthesized UCNPs in cyclohexane, (ii) UCNP-cement powder under 980 nm excitation, and (iii) a custom-design setup utilized for in situ emission measurements of UCNP-cement samples with a commercial spectrofluorometer. The laser beam excites a circular spot with a diameter of 2 mm. The cement samples had a thickness of 1.5 mm.

Time-resolved luminescence measurements yielding the luminescence decay kinetics of the dispersed UCNP and the UCNP-cement samples were also performed with the Edinburgh Instruments Model FLS980-xD2-stm spectrofluorometer equipped with an electrically pulsed 8 W 978 nm laser diode (long square pulses, pulse width of 150 μs). The decay kinetics were recorded at 540 (green Er-UCL), 654 (red Er-UCL), and 1000 nm (Yb-DCL), by using time-correlated single photon counting (TCSPC). The luminescence lifetimes were calculated from the measured decay kinetics with the FAST software (Edinburgh Instruments) by using a second-order exponential decay fit. The decay curves of the long-lived UCL were used as obtained without consideration of the instrument response function (tail fit, no unfolding of the instrument response function).

### X-ray Diffraction (XRD)

XRD measurements were conducted on a BRUKER D2 Phaser (second Gen) instrument using Cu Kα radiation (λ = 1.5419 Å) at 30 kV and 10 mA with the LYNXEYE XE-T detector using 2.5° secondary Soller slits. The primary Soller slit was set to 2.5°, and the divergence slit to 1.0 mm (equivalent to 0.95°). The step size was 0.02° 2θ with a measurement time per step of 1 s. XRD measurements were performed with UCNP-cement pastes at 6, 24, and 48 h after mixing. Therefore, sample hydration was stopped using the solvent replacement method.^29,30^ Data analysis was performed using the Match! software.

### Isothermal Heat-Flow Calorimetry

Calorimetric measurements were performed using a TAM Air isothermal calorimeter (TA Instruments). Sample preparation involved the mixing of cement (14.0 g) and deionized water (7 g) for 60 s at 200 rpm and for another 60 s at 400 rpm by using an IKA STARVISC 200-2.5 mixer. The stirrer was a stainless-steel loop with a rod diameter of 5 mm. From this mixture, samples were taken for calorimetric measurements (about 14 g). All test measurements were conducted at 20 °C with three independently prepared samples for each cement paste mixture.

## Results and Discussion

To assess the applicability of multicolor emissive lanthanide particles for the monitoring of early stage cement hydration processes and to gain first insights into their principal suitability for cement tagging, we determined the evolution of the luminescence properties of self-made 25 and 55 nm-sized core-only NaYF_4_:Yb,Er UCNPs of varying surface chemistry and commercial μm-sized Y_2_O_2_S:Yb,Er particles (referred to here also as UCNPs for simplicity reasons) in the first hours of cement hydration, with its four characteristic stages. Stages covered included (I) the initial period, in which the cement constituents dissolve and calcium silicate hydrates (C–S–H) and reaction phases from calcium aluminates and calcium sulfates such as ettringite (AFt) are rapidly formed, (II) the induction period, in which the initially rapid dissolution precipitation slows down significantly and recrystallization of sulfate aluminate phases takes place, (III) the acceleration period, in which calcium silicate hydrates are formed, and (IV) the deceleration period, in which the reactions slow down due to constricted crystal growth and more complex diffusion processes. As summarized in Figure 1, parameters screened from the UCNP reporter side included particle size, morphology, surface chemistry, luminescence features, and concentration in the respective cement mixtures. From the cement side, the influence of the chemical, i.e., mineral composition of the cement matrix and the water/cement ratio were examined. As inorganic carbon and silica nanoparticles have previously been shown to influence cement properties and hydration kinetics,^31,32^ in addition to optical methods such as reflection and luminescence spectroscopy, suitable for online *in situ* monitoring, we employed XRD (powder X-ray diffractometry) and isothermal heat-flow calorimetric measurements to determine whether the incorporation of UCNPs could impair cement hydration processes.

With this method combination, we aimed to identify suitable lanthanide particle reporters, here with focus on size and surface coating for the application in cementitious materials, and to determine possible reporter influences on cement hydration kinetics, thereby deriving suitable conditions to minimize UCNP concentration effects while preserving a high level of information content. In addition, reporter stability studies, covering the time frame of the cement hydration processes explored, were done over a period of about 18 months, which are still ongoing, to assess the future applicability of such lanthanide nanoparticles as authentication tags for construction materials.

### Preparation of UCNP-Doped Cement Samples

To lay the ground for the intended screening study of UCNPs reporter for cement tagging, we developed a procedure for the preparation of particle-doped cement samples and pastes for the subsequent optical studies that ensured a homogeneous distribution of the UCNPs in the starting materials forming the cement paste. For the development of this procedure, we exemplarily chose the smallest UCNPs of our reporter series with the highest surface-to-volume ratio, i.e., 25 nm OA capped core-only UCNPs, the luminescence pattern and intensity of which are expected to be particularly affected by the particle environment, especially by near surface quenchers.^33^ In addition, these UCNPs can be easily prepared in relatively large amounts of up to 5 g of monodisperse particles with a very high reproducibility of size, shape, crystal phase, chemical composition, and photoluminescence properties,^7^ and have been previously utilized by us for stability studies assessing different UCNP surface coatings.^34^ For the sample preparation, self-made OA coated 25 nm NaYF_4_:Yb­(20%),Er­(2%) UCNPs were ground together with cement powder of OPC for 5 min using a mortar and a pestle. Steady state photoluminescence measurements, performed with a 980 nm laser as excitation light source, revealed the characteristic sharp and intense emission bands of Er^3+^ present in these UCNPs in the green and red region of the visible spectrum in this cement mixture (Figure 2a and b). To ensure a homogeneous distribution of the UCNPs in the cement mixture, we measured the UCL spectra under identical measurement conditions at different locations within the cement paste. The excellent match of these spectra shown in the Supporting Information (SI) in Figure SI1 confirms the homogeneity of the UCNP distribution and, hence, the suitability of our mixing procedure. The environment sensitivity of the UCNP UCL and environment-induced changes in the UCNP emission pattern are highlighted in Figure 2a and b, comparing the emission spectrum of the UCNPs in the cement paste with the luminescence spectra of the OA capped UCNPs in a nonpolar organic solvent such as cyclohexane and the ligand-free UCNPs in water, obtained after removal of the hydrophobic OA surface ligands through a treatment with HCl.^10^ As follows from the luminescence spectra shown in Figure 2a, which are normalized at the strongest emission band of the particles, respectively, which is at 540 nm (green) for the oleate-capped particles and at 654 nm (red) emission band of Er^3+^ for the ligand-free particles. The more pronounced red emission of the UCNPs relative to the green emission in the presence of water originates from the commonly observed quenching of the luminescence of such core-only UCNPs by near surface water molecules.^28,35^ This is a result of an increased nonradiative relaxation of the ^4^I_11/2_ to the ^4^I_13/2_ energy level, caused by the high energy vibrational modes of near surface water molecules.^36^

**Figure 2:**
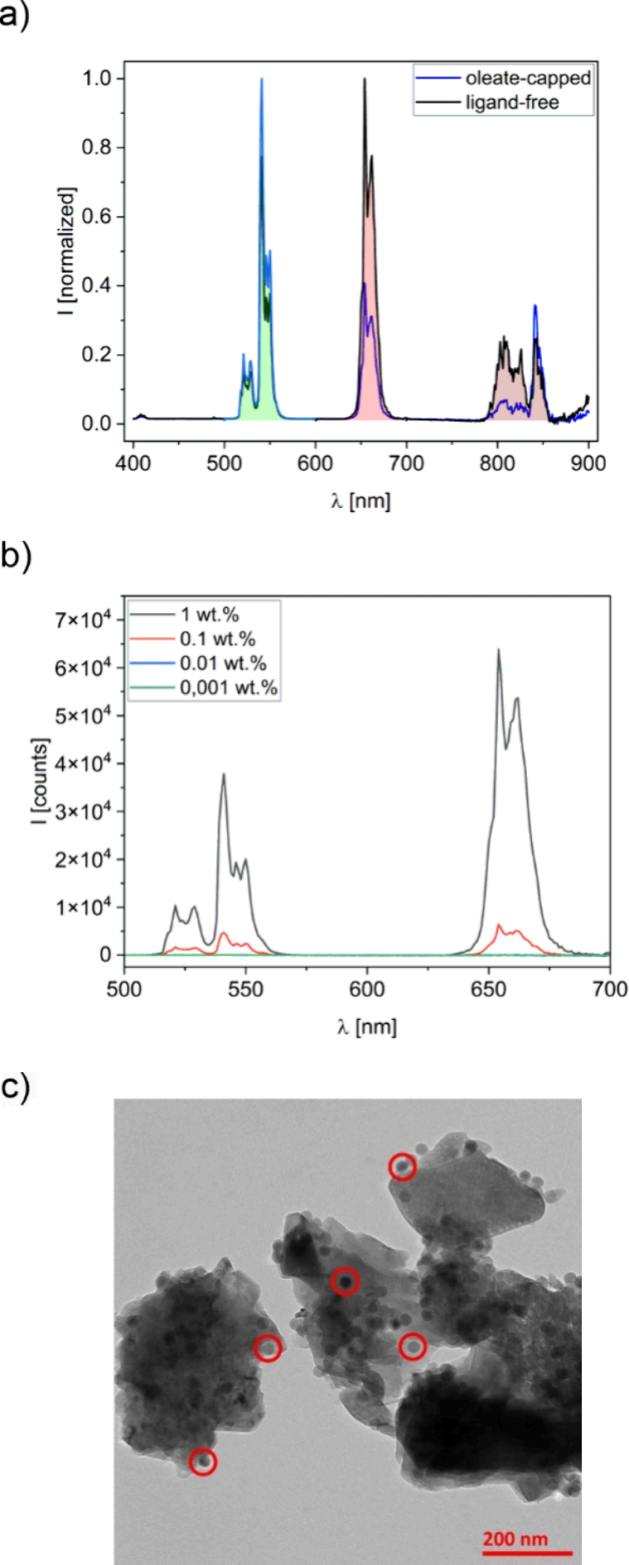
**a)** Emission spectra of 25 nm oleate-capped UCNP dispersed in cyclohexane and ligand-free UCNP dispersed in water measured with a spectrofluorometer, excited at 980 nm and normalized at their respective strongest emission bands, 540 nm for the oleate-capped particles in cyclohexane and 654 nm for the ligand-free particles in water, to visualize the differences in band ratios originating from the surface chemistry and UCNP (micro)­environment. **b)** Concentration-dependent emission spectra of 25 nm oleate-capped UCNPs in dry OPC powder determined for UCNP concentrations of 0.001, 0.01, 0.1, and 1 wt %. **c)** TEM image of 25 nm oleate-capped UCNPs in OPC, prepared by drop casting a dispersion of UCNP-tagged OPC in cyclohexane on the TEM grid, revealing that the UCNPs are dispersed all over the cement granules. Selected UCNPs are marked with red circles.

Subsequently, we determined the limit of detection (LOD) for the photoluminescence (UCL) of these 25 nm oleate-capped UCNPs in the dry cement powder mixtures of OPC for UCNP concentrations of 1, 0.1, 0.01, and 0.001 wt % using identical instrument settings and a low excitation power density of 7.07 W/cm^2^. The UCL intensities resulting for the three different UCNP concentrations are proportional to the particle concentration in the OPC paste. This points to the absence of interactions of the UCNPs with the dry cement matrix. As revealed in Figure 2b under these conditions, UCNP concentrations as low as 0.1 wt % can be detected in dry OPC. Even though lower UCNP concentrations are in principle detectable by increasing the excitation power density of the 980 nm laser, this could lead to a considerable heating up of the cementitious materials due to the absorption of water molecules at 980 nm. As the luminescence features of UCNPs are temperature sensitive, which is often exploited for temperature sensing, reading out, e.g., the intensity ratios of the two green Er^3+^ emission bands at 525 and 540 nm, which are in thermal equilibrium,^37^ a temperature increase could affect the spectral distribution and intensity of the UCNP UCL emission. This could distort the desired information about the cement hydration processes. In addition, as shown in Figure 2c, the incorporation of the UCNPs into the OPC matrix was confirmed by TEM. These TEM images revealed an adsorption of the UCNPs onto the surface of the cement granules and demonstrated the preservation of UCNP size and shape after the grinding process and contact with the cementitious environment. As revealed by these TEM images, the UCNP particles are well separated on the surfaces of the cement granules, with no hint of particle aggregation. We also did not notice aggregation of the UCNPs in the dry cement powder after grinding.

Next, to determine whether the UCNP incorporation could affect the kinetics of the cement hydration, possibly in a UCNP-type and concentration dependent manner, we performed isothermal heat-flow calorimetry measurements with UCNPs of varying size, surface chemistry, and concentration in OPC. This included 25 nm oleate-capped UCNPs in concentrations of 0.1 and 1 wt % as well as 25 nm ligand-free UCNPs and 55 nm UCNPs, capped with oleate and ligand-free at concentrations of 1 wt %. The corresponding cumulative heat and heat flow curves of the UCNP-cement samples are displayed in Figure 3a and Figure 3b and compared with the hydration kinetics of pure cement, i.e., OPC. As follows from the cumulative heat and heat flow curves of the UCNP-cement samples summarized in Figure 3a and Figure 3b, for all UCNPs assessed, the addition of 1.0 wt % of UCNPs notably slows down the cement hydration, as is indicated by the prolongation of the induction period, but to a different extent. The hydrophobic, oleate-capped 25 nm UCNPs retarded hydration more strongly compared to ligand-free UCNPs of a similar size, pointing to an influence of particle surface chemistry. Also, UCNP size seems to matter, as larger 55 nm-sized UCNPs added in a concentration of 1.0 wt % introduce a significantly reduced retardation effect. A comparison of 55 nm-sized oleate-capped and ligand-free UCNPs also reveals a retardation effect of the hydrophobic oleate surface ligands, as observed for 25 nm UCNPs. Please note that the presence of UCNPs, regardless of size, surface chemistry, and concentration, does not change the character of the heat flow curve, beyond the retarded acceleration period. Only marginal changes can be observed for the maximum of the heat flow peak, which is slightly higher for the smaller sized UCNPs. This finding confirms that the UCNPs do not disturb the sulfate-aluminate balance but rather interfere with the dissolution and precipitation of the silicate and calcium phases and Ca­(OH)_2_. We assume that the UCNPs may retard the hydration process similarly to sugars by adsorbing to the cement particle surfaces, forming temporary barriers to hydration. The adsorption of the UCNPs to the cement was confirmed by TEM as shown in Figure 2c. Furthermore, a possible release of oleate-ligands from the UCNP surface during hydration could introduce more chelating agents into the mixture.

**Figure 3:**
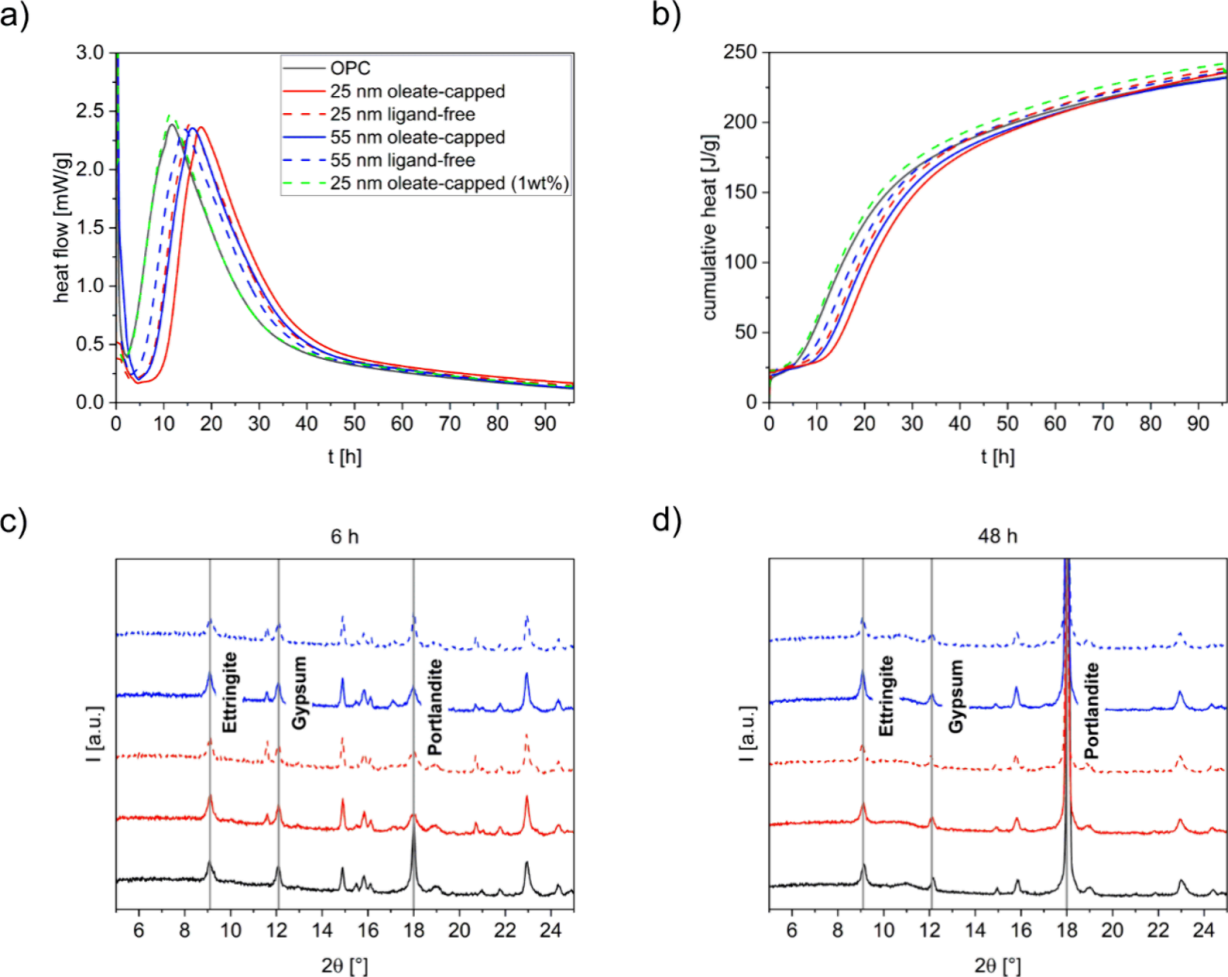
**a)** Heat flow curves and **b)** cumulative heat curves of UCNP-cement samples made from 25 and 55 nm oleate-capped (solid lines) and ligand-free (dashed lines) UCNPs added in a concentration of 1 wt %, compared to a UCNP-cement sample containing 0.1 wt %, of 25 nm oleate-capped UCNPs; X-ray diffractograms of the UCNP-cement samples containing 1 wt % of 25 or 55 nm oleate-capped and ligand-free UCNPs measured after **c)** 6 h, and **d)** 48 h of cement hydration.

The slight retardation observed for a UCNP concentration of 1.0 wt % can be effectively reduced by decreasing the UCNP concentration to 0.1 wt %. At this concentration, no delay could be observed, and the maximum heat flow peak seems to be even slightly increased. The cumulative heat flow curve highlights the retarded hydration kinetics of the UCNP-containing samples during the first 12 h. Later, even the UCNP cement sample, containing a concentration of 1.0 wt % of 25 nm oleate-capped UCNPs, which reveals the strongest retardation, undergoes a significant acceleration of the hydration process. After 96 h, all samples exhibit similar total heat values. Since the heat flow evolution typically corresponds to strength development, it can be assumed that the mechanical properties of the cement material are not affected by the presence of the particles. These findings underline the principal suitability of the assessed UCNPs for cement probing.

The XRD patterns shown in Figure 3c and Figure 3d, which were measured after 6 and 48 h, display the impact of the UCNPs differing in size and surface chemistry on the evolution of the OPC phases for UCNP concentrations of 1.0 wt %. After 6 h, all samples contained a considerable amount of ettringite (Ca_6_Al_2_(SO_4_)_3_ (OH)_12_·26H_2_O), which is the main product of the reaction between C3A, sulfates, and water. The presence of UCNPs leads to a delay of the portlandite formation process as suggested by the smaller amount of portlandite formed. These findings confirm the results of the heat flow experiments, which point to an extended induction period in the presence of the UCNPs with a consequently delayed accelerated period. Regardless of UCNP surface coating, both samples containing 25 nm UCNPs reveal a notably reduced amount of portlandite compared to the samples containing 55 nm UCNPs and the neat cement paste. As the availability of portlandite is necessary for the accelerated C–S–H hydration at the end of the induction period, which is in line with the observation of a reduced retardation effect for UCNP cement samples made from 55 nm UCNPs compared to samples containing 25 nm UCNPs. After 48 h of hydration, all samples reveal the same amount of portlandite, supporting the data obtained by isothermal calorimetry. The XRD pattern show no major differences between oleate-capped and ligand-free particles of the same size.

In addition, all UCNP cement samples exhibit prominent gypsum peaks in contrast to those of the UCNP-free cement paste used as a control. The presence of gypsum points to an early interaction of the UCNPs affecting the dissolution of gypsum and/or its consumption, yielding ettringite and monosulfate phases. The soluble gypsum phases from the set retarder, which are typically a compound of anhydrite, hemihydrate, and dihydrate, react with the calcium aluminate phases (C3A) to predominantly form ettringite and some monosulfate phases at an early stage. Excess soluble calcium sulfates or calcium aluminates can cause the formation of either secondary gypsum (the hemihydrate reacts with water to form the dihydrate) or calcium aluminate hydrates (C-A-H). Apparently, the UCNPs hinder gypsum from solvating or aluminates from interacting with the gypsum phases. Given the expected electrostatic interactions between the aluminate phases and the UCNP, we hypothesize that the adsorption of the UCNPs on the aluminate phases hinders the interactions between sulfates and aluminates and assume that the gypsum peak is a result of secondary gypsum formation. This effect could possibly also occur at the onset of the slightly increased maximum heat flow curve. The presence of gypsum and the reduced amount of portlandite formed in the presence of UCNPs support retardation of the aluminate and silicate reactions by the UCNPs. As indicated by the XRD studies, the reduced retardation observed for the UCNP-cement sample containing 55 nm UCNPs compared to the UCNP-cement sample containing 25 nm UCNPs is mainly caused by the enhanced formation of portlandite. According to the cumulative heat release, after 24 h, there is still a prominent difference between the samples containing 1.0 wt % of UCNP. In terms of ettringite and portlandite formation, however, the difference between cement samples containing 55 and 25 nm UCNPs becomes less prominent. After 48 h of hydration, the cumulative heat release reached an identical range for all UCNP cement samples. This follows from the same amount of portlandite that was obtained.

### Exploring the Luminescence Properties of UCNPs in Cement Pastes in the Early Hydration Phase

To explore the potential of 25 nm oleate-capped UCNPs for cement probing, different amounts of water (5.5–16.5 mg) were added to 22 mg of our OPC powder mixture containing UCNPs. After water addition, the emission spectra of the UCNP-cement samples were acquired every 5 min over a period of 24 h, using the custom-designed spectroscopic setup shown in Figure 1. This miniaturized setup was previously designed by us for optical studies of cement hydration with organic dyes and validated.^21^ For the evaluation of the changes in the UCL intensity during the early phase of cement hydration, we focused on the green and red emission bands of Er^3+^ located at 525, 540, and 654 nm. Thereby, the peak areas under the three strongest emission bands were integrated and plotted as a function of time (Figure 4a). The UCL bands in the near-infrared (NIR) at wavelengths above 800 nm were not further examined, because of the strong background in this wavelength region. Moreover, the intensity ratio of the two green Er^3+^ emission bands was monitored to determine whether heating effects occur due to laser excitation at 980 nm at the chosen relatively low excitation power density in the presence of water which also absorbs at this wavelength. Thereby, strong heating effects could be excluded. To compromise between a sufficiently high emission intensity at low excitation power densities and a small influence on the cement matrix, a concentration of 1 wt % of UCNP was used for all further experiments.

**Figure 4:**
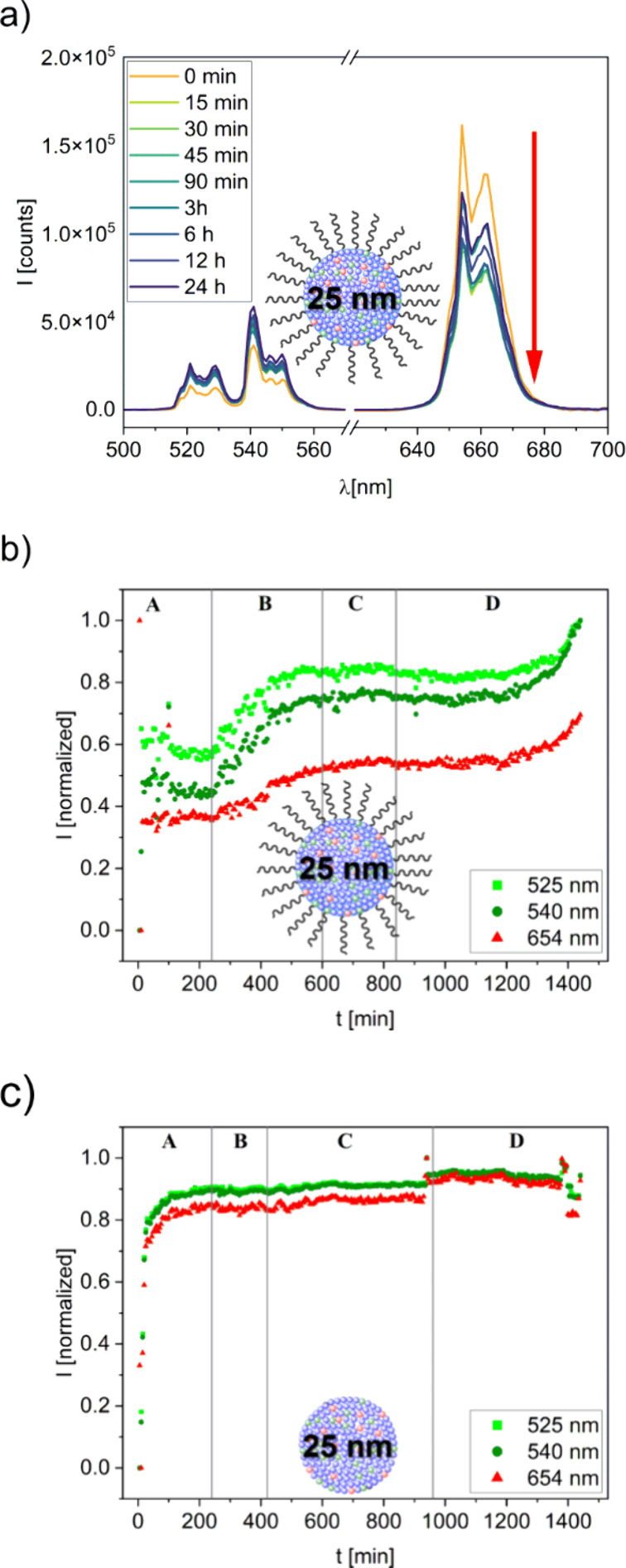
**a**) Emission spectra of 25 nm oleate-capped UCNPs in OPC (1 wt %) and integrated emission intensities of the green and red emission bands during (A) the induction period, (B) the acceleration period, and (C) and (D) the retardation period obtained for **b)** 25 nm oleate-capped UCNPs and **c)** ligand-free UCNPs utilizing concentrations of 1 wt %, respectively. The water/cement ratio was 0.5. Luminescence excitation was performed at 980 nm.

### Assessing a Possible Influence of UCNP Surface Chemistry

The interaction of nanoparticles with their microenvironment is largely determined by the particle surface, i.e., surface coatings and functional groups, which also determine the particle dispersibility. In the case of UCNPs with their luminescence being prone to surface quenching, e.g., by quenchers such as molecules with high energy vibrational modes like O–H or N–H groups,^35^ particularly for core-only particles lacking thick surface protection and passivation shells,^9^ the UCNP microenvironment can also influence the UCL characteristics.^33^ For example, the sensitivity of UCL to such quenchers has been exploited for sensing the presence of water molecules. To complement the previous studies on the compatibility of oleate-capped and ligand-free UCNPs with the cement matrix and to derive a possible influence of the surface chemistry of UCNP particles on their luminescence characteristics in cementitious media, we exemplarily compared the UCL properties of 25 nm oleate-capped and ligand-free UCNPs during the early hydration phase for UCNP concentrations of 1 wt %. The integrated emission bands obtained within the first 24 h of hydration are displayed in Figure 4b and c showing that both UCNPs reveal a similar luminescence behavior in the cement pastes.

As revealed in Figure 4a, the UCNP emission pattern changes over the course of cement hydration. In the first minutes after water addition, both UCNPs experience a sharp drop in their emission, coinciding with the initial period of the cement hydration. In this early hydration phase, the cement constituents start to dissolve, leading to a drastic increase in the concentration of free ions, especially Ca^2+^, SO_4_^2–^, AlO_4_^5–^, and SiO_4_^4–^. This is apparently responsible for the observed significant surface quenching and the rapid decrease in UCL intensity. After this short initial period, the integrated emission intensities experience a strong rise for the first 90 min, followed by a phase of slight increases extending over 24 h. The optical properties of the UCNPs are apparently stable in the harsh cementitious environment within the time of the measurement, which, in turn, suggests the absence of particle disintegration. Moreover, while the emission intensities change, the ratio of the two green Er^3+^ emission bands remain constant during the luminescence measurements, confirming the absence of heating effects for the chosen measurement conditions. However, for the oleate-capped particles, the increase in emission intensity during the initial period is not as steep and fast as observed for the sample containing 25 nm ligand-free UCNPs, as revealed in Figure 4b and Figure 4c.

As previously shown by stability studies of UCNPs with different surface coating in different environments, the luminescence decay kinetics of NaYF_4_:Yb,Er UCNPs are particularly sensitive to changes in the particle environment and particle surface chemistry.^1^ Therefore, for the 25 nm OA-capped and ligand-free UCNPs, we also examined the luminescence decay kinetics of the green Er^3+^ emission band at 540 nm. Representative fluorescence decay measurements reveal similar lifetimes of both UCNPs amounting to τ = 121 and τ = 113 μs for oleate-capped and ligand-free particles in the beginning of cement hydration (SI, Figure SI 4). Within the first 12 h of hydration, the luminescence decay times significantly decrease to values of τ = 87 μs and τ = 88 μs for the oleate-capped and the ligand-free particles. Then, the luminescence decay kinetics and lifetimes remain constant. These findings, together with the results of the steady state emission measurements underline the considerable changes in UCNP luminescence mainly in the first 1.5 h of hydration. At longer hydration times, the UCNP luminescence reaches constant values. The closely matching UCL lifetimes of both particle samples indicate a similar chemical environment of the UCNPs directly at the surface, pointing to a possible removal of the oleate surface ligands in the cement matrix, with the hydrated cement subsequently coating the surface of the UCNPs. The more complex evolution of the emission intensities and the slightly more pronounced changes in the luminescence lifetimes of the 25 nm oleate-capped UCNPs suggest that these UCNPs are more responsive to changes in the OPC matrix.

### Influence of Particle Size and Water to Cement Ratio on UCNP Emission Kinetics in OPC

Next, to examine a possible influence of UCNP size and morphology on the luminescence monitoring of cement hydration with UCNP reporters, we performed similar *in situ* studies for self-made 55 nm rod-like shaped oleate-capped UCNPs. These particles were obtained by the same general procedure as the 25 nm particles by lowering the OA-to-ODE ratio to 2:8. As follows from Figure 5a and b, comparing the time courses of the UCL pattern and integral intensities determined with 25 and 55 nm oleate-UCNPs, the emission spectra acquired *in situ* with both types of UCNPs strongly differ. For the 55 nm rod-like shaped UCNPs, a strong decrease in emission intensity occurs upon the onset of cement hydration that asymptotically approached zero, i.e., complete luminescence quenching. This effect is tentatively ascribed to the decomposition or mechanical degradation of the 55 nm UCNPs leading to a complete loss in luminescence. Apparently, particle size and/or shape make these 55 nm rod-like shaped particles more prone to decomposition by mechanical stress like shear forces than the relatively stable 25 nm sized, spherical UCNPs. The mechanical strength of the nanoparticles is reportedly a function of both their size and shape. While not explicitly proven for UCNPs, similar nanoparticle systems generally show a higher stability against mechanical stress the smaller they are and the more spherical they are.^38,39^ As shown by us and others before, the decomposition of UCNPs leads to a loss in emission intensity and a change in fluorescence decay kinetics and lifetimes.^34,40,41^

**Figure 5:**
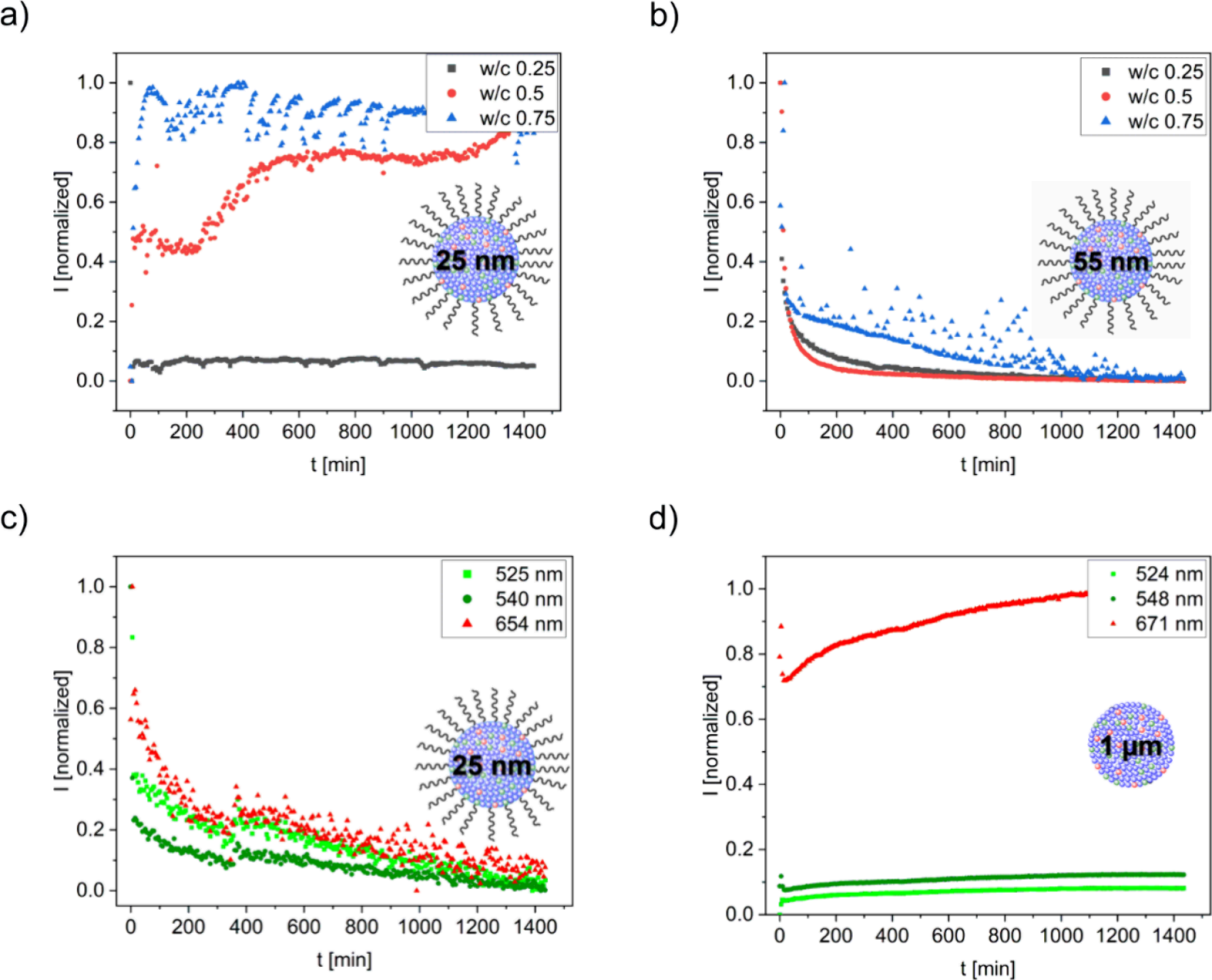
Top: Normalized, integrated emission intensities of the 540 nm emission bands obtained for **a)** 25 nm UCNPs and **b)** 55 nm UCNPs in the first 24 h of cement hydration for different water-to-cement (w/c) ratios. Bottom: Normalized, integrated emission intensities derived for the green and red emission bands for **c)** 25 nm oleate-capped particles in sulfate resistant cement and for **d)** ligand-free commercial upconversion microparticles in OPC. For all samples, a UCNP concentration of 1 wt % was utilized. Luminescence excitation was at 980 nm.

To further study size effects, we extended our study to representatively chosen commercial μm-sized upconversion particles, here ligand-free Y_2_O_2_S:Yb,Er microparticles. The evolution of the green and red emission bands of these microparticles during early hydration is shown in Figure 5d. The emission spectrum of the neat particles is displayed in the SI in Figure SI 5. The Y_2_O_2_S:Yb,Er microparticles show emission maxima at 524, 548, and 671 nm upon excitation at 980 nm, that slightly differ from the emission spectra of the self-made UCNPs with a fluoride host matrix. As follows from Figure 5d, the emission intensity of the ligand-free Y_2_O_2_S:Yb,Er microparticles in the cement sample strongly decreases during the first 30 min of hydration before asymptotically approaching a maximum emission intensity after 24 h. These effects are ascribed to changes in the microparticle surface chemistry and environment and particle decomposition by mechanical degradation as reasoned before for the UCNPs.^34,38–41^ Hence, for further studies on the suitability of lanthanide particles for the optical probing and monitoring of cement hydration processes and first screening studies on the possible applicability of such particles for cement tagging, we mainly focused on self-made 25 nm oleate-capped UCNPs.

As the hydration and setting and therefore the mechanical properties of concrete are highly dependent on the water to cement (w/c) ratio, the influence of different water-to-cement ratios (w/c) on the emission characteristics of 25 and 55 nm oleate-capped UCNPs was determined. For both particle sizes, for high w/c, the time required to reach a constant luminescence is increased. For w/c = 0.75, a constant emission of the 25 nm UCNPs is observed after about 8 h, compared to 60 min for w/c = 0.5 and 30 min for w/c = 0.25. The emission of the 55 nm UCNPs asymptotically approaches complete luminescence quenching after approximately 7 h for w/c = 0.25 and 0.5, while the slope of the decay observed for w/c = 0.75 is significantly less steep, and complete luminescence quenching occurs at 20 h of hydration. High w/c ratios generally lead to a more porous cement paste upon hardening, which would allow for a larger number of UNCPs per pore. Under these conditions, possible mechanical effects influencing the particle morphology, like shear forces or pressure through growing crystal phases, seem to be delayed or less pronounced. As revealed by this study, generally, the use of smaller UCNPs seems to be beneficial considering their stability and lack of complete loss in luminescence, which compensates for their lower brightness.

### Influence of Cement Constituents

As cement is a mixture of several different chemical components, the cement hydration process itself is the sum of different chemical reactions and processes. To gain a better understanding of the influences of the hydration processes of the different cement constituents and different cement compositions on the emission behavior of the UCNP reporters, we performed *in situ* emission experiments with oleate-capped and ligand-free 25 nm UCNPs and the pure main cement constituents tricalcium aluminate (C3A) and tricalcium silicate (C3S), gypsum, as well as with sulfate resistant cement (Figure 6). The resulting changes in luminescence properties were then compared to the effects previously described for the OPC. These experiments reveal a strikingly different behavior of the UCNP emission for the two pure cement constituents. In C3S, which is the main clinker phase, the 25 nm UCNPs exhibit a time-dependent luminescence behavior, comparable to that observed in OPC, i.e., a strong increase in emission during the first minutes of hydration followed by a slow and continuous decrease in luminescence within the first 24 h of hydration. Hence, the observed major changes in UCNP emission intensities are attributed to the induction period of C3S hydration.

**Figure 6:**
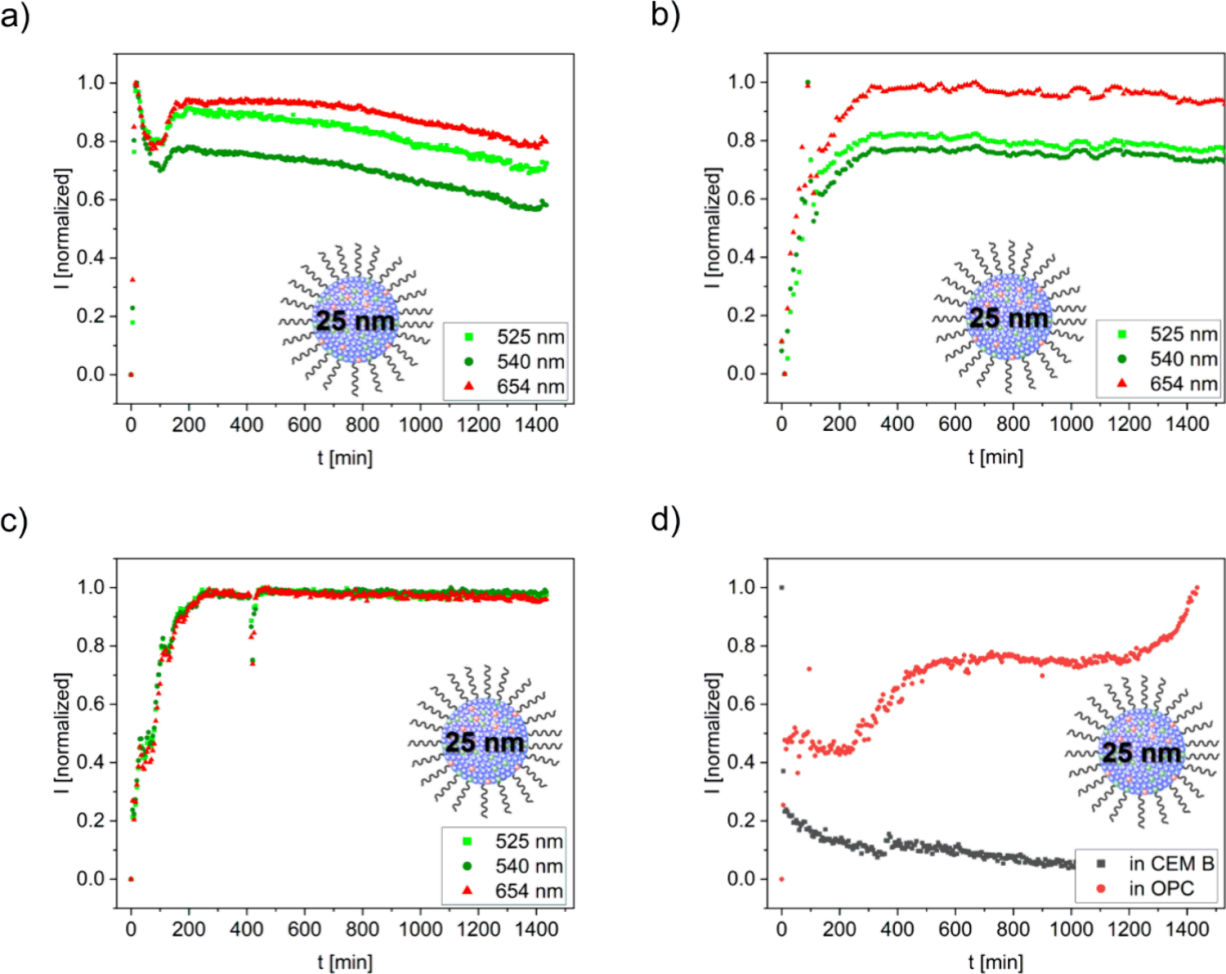
Normalized, integrated green and red emission intensities (525 nm; 540 nm; 654 nm) of 25 nm oleate-capped UCNPs obtained in the first 24 h of cement hydration for **a)** C3S, **b)** C3A, and **c)** gypsum. **d)** Comparison of the evolution of the 540 nm emission band of 25 nm UCNPs in sulfate-resistant cement CEM B and OPC. Luminescence excitation was at 980 nm.

As OPC is composed of 50–75% C3S and only 5–15% C3A,^42^ C3S hydration seems to dominate the observed UCNP emission behavior compared to C3A hydration, even though the UCNPs reveal a stronger luminescence in the pure aluminate phase. To confirm this hypothesis, we performed *in situ* hydration experiments with sulfate-resistant cement. For this type of cement, the UCNP emission follows the same trend as observed for the pure C3S phase, with a significant increase in the initial hydration phase and a reduction over the course of 24 h. Qualitatively, these luminescence changes present an inversion of the time-dependent luminescence effects observed for OPC. However, as the sulfate resistant cement contains less than 5% C3A, the effect of C3S hydration on the time dependence of the UCNP luminescence is dominant. This accounts for the similarities between the pure phase and the sulfate resistant cement. In contrast, the luminescence characteristics of the UCNPs in C3A during the initial period of hydration are closely match with those observed in OPC in the same time frame. This suggests that the aluminate reaction is mainly responsible for the observed changes in UCNP emission.

### Long-Term Stability Studies - Exploring the Potential of UCNPs as Cement Tags

To gain first insights into the applicability of UCNPs for the tagging of cement, e.g., for anticounterfeiting applications, long-term stability studies with cement pastes containing 25 nm oleate-capped UCNPs were performed over a period of 18 months. These long-term stability studies reveal that the 25 nm UCNPs retain their emissive properties in cement over a period of 1.5 years after the initiation of cement hydration. The UCL intensities of the UCNPs decrease by a factor of 2 × 10^4^ compared to the initially obtained luminescence intensities, as shown by the weak luminescence signal in Figure 7a, which is nevertheless still easily detectable even at the chosen low excitation power density and the previously optimized measurement conditions. These effects are largely ascribed to a significant increase in the optical density of the hardened cement, possibly flanked by the decomposition of some particles in the cement matrix. Time-resolved luminescence studies demonstrate a strong reduction in the luminescence lifetime of the 540 nm emission band of Er^3+^ from initially τ = 87 μs to τ = 53 μs for the aged sample and a tailing of the luminescence decay profile yielding a lifetime τ of 133 μs; see Figure 7b. The change in the luminescence decay kinetics with a prominent tailing at longer times of hydration is ascribed to the back energy transfer from Er^3+^ to Yb^3+^, which is especially prominent in strongly quenched samples.^34^ Even though the particle brightness is low after several months and the luminescence lifetimes point to considerably quenched particles, the UCNP emission was nevertheless still easily detectable. This supports the applicability of UCNPs for cement tagging and hydration monitoring over extended time frames.

**Figure 7:**
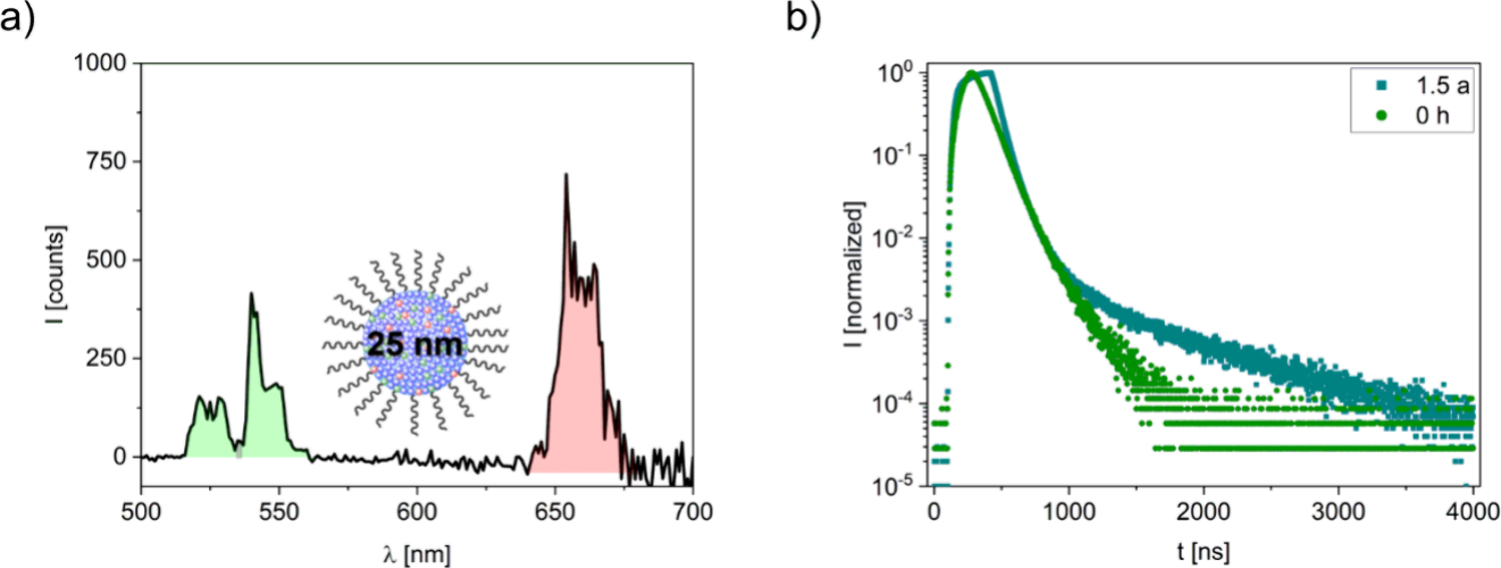
**a)** Emission spectrum of 25 nm UCNPs in cement after 1.5 years; **b)** comparison of the luminescence decay kinetics of the aged and a freshly prepared UCNP cement sample detected at 540 nm. Excitation was at 980 nm.

Recently performed first leaching studies with nonhardened and hardened cement pastes containing lanthanide nano- and microparticles with cyclohexane did not provide a hint for a possible removal of these particles in either TEM or fluorescence measurements. Apparently, the particles are already tightly bound to dry cement. Release of maximally very small amounts of lanthanide particles into the environment can most likely only occur due to mechanical degradation of the respective cementitious material. Toxicity studies with lanthanide nanoparticles performed by us and other research groups reveal a relatively low toxicity of these materials and their constituents,^43^ with most likely fluoride ions being the components with the highest potential toxicity,^44^ however, for concentrations that exceed the ones which are expected to be maximally released from our lanthanide particle-stained cementitious materials.

## Conclusion and Outlook

In this work, by screening the luminescence properties of differently sized lanthanide-based core-only upconversion nanoparticles (UCNPs) of varying surface chemistry in cement pastes, we could demonstrate the applicability of UCNPs as luminescent probes for monitoring the early stages of cement hydration. The observed changes in the spectral distribution and intensity of the luminescence of lanthanide nano- and microparticles reflect the coupling of the O–H vibrations from near surface water molecules to the ^4^S_3/2_/^2^H_11/2_ and ^4^I_11/2_ energy levels of Er^3+^ and the corresponding increase in nonradiative decay rates of these levels and changes in size due to mechanical decomposition. This quenches the Er^3+^ luminescence and favors red emission. Such luminescence probing studies cannot provide more selective information on specific processes in complex cementitious systems.

The upconversion luminescence (UCL) of representatively chosen NaYF_4_:Yb,Er nanoparticles in ordinary Portland cement (OPC) is detectable for concentrations as low as 0.1 wt % for the smallest 25 nm UCNPs assessed. The luminescence features of the UCNPs in cement paste and the time-dependent evolution of the UCL intensity and pattern reveal a considerable impact of particle size and morphology. During the first 24 h of cement hydration, the luminescence properties of the UCNPs considerably changed, depending on UCNP size and particle surface chemistry. Generally, oleate-capped UCNPs more strongly respond to the different phases of early cement hydration than ligand-free particles. Spherical 25 nm sized particles show an increase in emission intensity, while the emission of rod-like 55 nm sized UCNPs quickly disappears during the first 24 h of hydration, indicating particle decomposition. A commercial, micrometer sized upconversion phosphor proved to be less responsive to changes in the cementitious environment than the UCNPs. The emission behavior of 25 nm UCNPs also significantly differed for the cement constituents, C3S, C3A, and gypsum, as well as sulfate-resistant cement, indicating a responsivity to the different chemical processes involved in the hydration processes. Isothermal heat-flow calorimetry measurements confirmed that the UCNPs do not change the character of the cement hydration, independent of UCNP size, morphology, surface chemistry, and concentration, but retard the involved processes within the first 24 h. This was supported by XRD studies, revealing a retarded formation of ettringite and portlandite for the UCNP-cement samples, compared to OPC due to a slowed down aluminate and silicate reaction. Overall, we could demonstrate that the addition of a small amount of 25 nm UCNPs of 0.1 wt % to cement has almost no effect on the cement hydration kinetics.

In addition, our long-term screening studies revealed the detectability of UCNP emission in UCNP cement samples after more than a year. This provides a clear hint for the applicability of such simple UCNPs for long-term probing and encoding of cement matrices. The latter could provide the basis, e.g., for UCNP tags for the control of material flows in cement supply chains or as anticounterfeiting or security barcodes, thereby exploiting, e.g., the composition control of the luminescence color of this class of nanomaterials. Although the reagent costs amounted to about 2 € for 1 g of UCNPs for the high purity lanthanide chloride starting materials used by us, which could be further reduced by employing less pure lanthanide ion salts, the use of this monitoring approach could be very advantageous for specialized applications, e.g., to study the hydration behavior of cement under different external conditions or comparing the effect of different chemical admixtures such as plasticizers, viscosity modifying agents, accelerators, and retarders on the hydration of cement. For the UCNP encoding of cementitious materials, e.g., also a localized, i.e., spatially restricted, encoding procedure could be employed to reduce material costs. Such studies are planned in the future.

As the cementitious environment and the underlying processes remain complex and the exact cause of the UCNP emission changes was not fully revealed, in the future we will focus on the detectability of UCNPs in cement also with other analytical techniques, which should be preferably suitable for online measurements such as XRF, with meanwhile relatively simple and inexpensive hand-held devices. With these correlative multimethod measurements, we aim for a deeper understanding of the processes responsible for the changes in UCNP emission properties during cement hydration. Additionally, the properties of the UCNP tagged cement under ambient conditions, such as weathering, freeze thawing, and carbonation, as well as in different cementitious systems, including additives such as grinding aids and superplasticizers, will be evaluated.

## Supplementary Material



## Notes

Additional analytical data including analytical characterization of UCNPs, XRD patterns and fit-parameters used for lifetime-measurements (PDF)

## Abbreviations

UCNP: Upconversion Nanoparticle
UV: ultraviolet
VIS: Visible
NIR: Near-infrared
SWIR: Short-wave infrared
OPC: Ordinary Portland Cement
C3A: Tricalcium aluminate
C3S: Tricalcium silicate
XRD: X-ray diffraction
wt %: Weight percent
UCL: Upconversion luminescence
DSL: Down-shifted luminescence
ODE: 1-Octadecene
RCF: Relative centrifugal force
TEM: Transmission electron microscopy
TCSPC: Time-correlated single photon counting
OA: Oleic acid
XRF: X-ray fluorescence
