# Supplementary material for: Assessing the Applicability of Lanthanide-Based Upconverting Nanoparticles for Optically Monitoring Cement Hydration and Tagging Building Materials
Source: ACS Omega. 2025 Jul 16;10(29):31587–99. doi: 10.1021/acsomega.5c02236 (PMC12311682; doi:10.1021/acsomega.5c02236)
Supplement: Supplementary file 1 [file ao5c02236_si_001.pdf]

Supporting Information

Assessing the applicability of lanthanide-based upconverting nanoparticles for optically monitoring cement hydration and tagging building materials

Philipp Kossatz,[1,4] *Alexander Mezhov,*[2] *Elina Andresen*[1], *Carsten Prinz,*[3] *Wolfram Schmidt,*[2,*]

and Ute Resch-Genger[1,*]

[1]: Division *Biophotonics*, Federal Institute for Materials Research and Testing (BAM), Richard-Willstaetter-Str. 11, D-12489 Berlin, Germany; ute.resch@bam.de

[2]: Division *Technology of Construction Materials*, Federal Institute for Materials Research and Testing (BAM), Unter den Eichen 87, D-12205 Berlin, Germany, wolfram.schmidt@bam.de;

[3]: Division *Structure Analysis*, Federal Institute for Materials Research and Testing (BAM), Richard-Willstaetter-Str. 11, D-12489 Berlin, Germany

[4] *Institute for Chemistry and Biochemistry*, Free University Berlin, Arnimallee 20, D-14195 Berlin, Germany

Contents

**Supporting Infomation**	**Page**
SI-1 - Analytical characterization of the UCNPs	S3
SI-2: Full powder XRD-patterns	S4
SI-3: Homogeneity of UCNP-cement samples	S6
SI-4: Photoluminescence lifetime-measurements and fit-parameters	S6
SI-5: Emission spectrum of commercial upconversion particles in OPC	S9

SI-1: Analytical characterization of the UCNPs

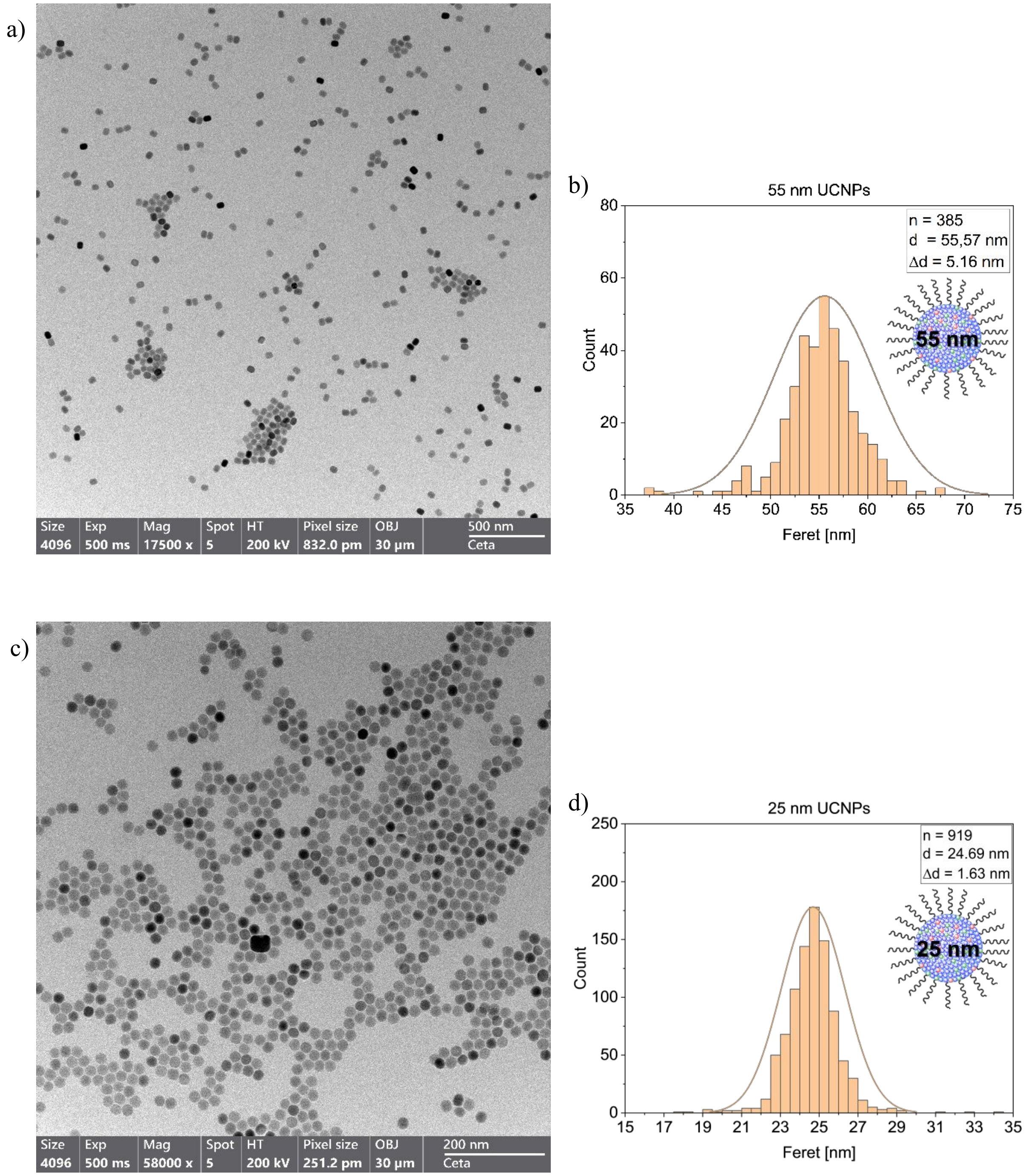

Figure SI 1: TEM images and corresponding size-histograms of synthesized a-b) 55 nm sized oleate-capped UCNPs and c-d) 25 nm oleate-capped UCNPs.

SI-2: Full powder XRD-patterns

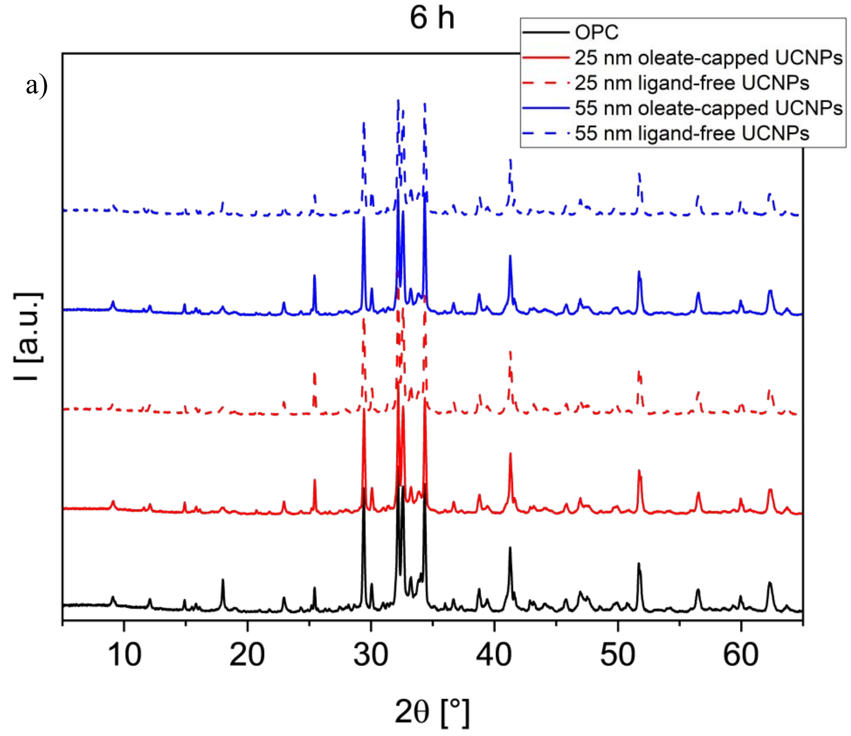

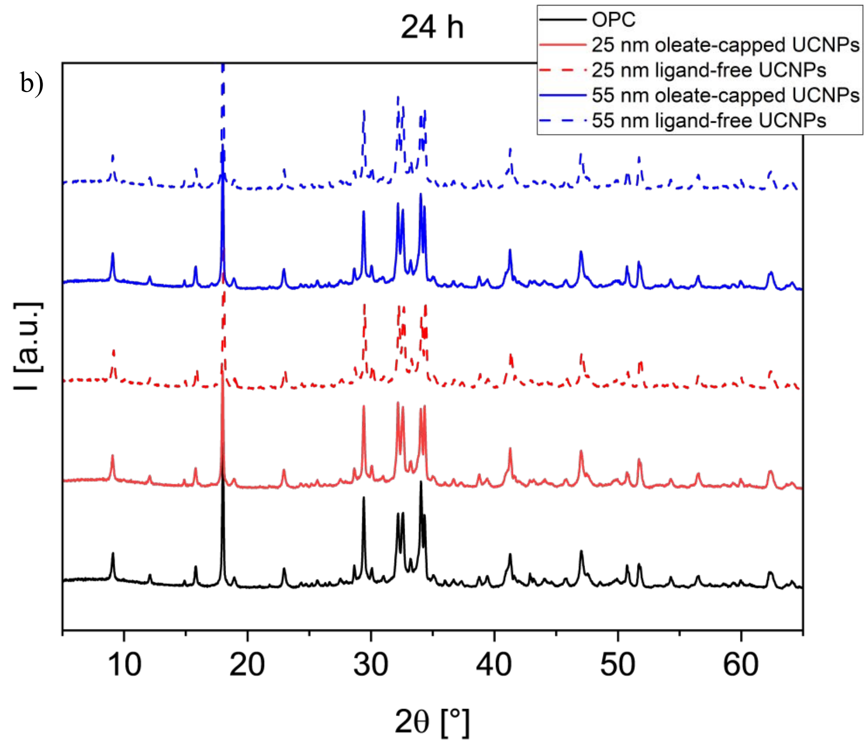

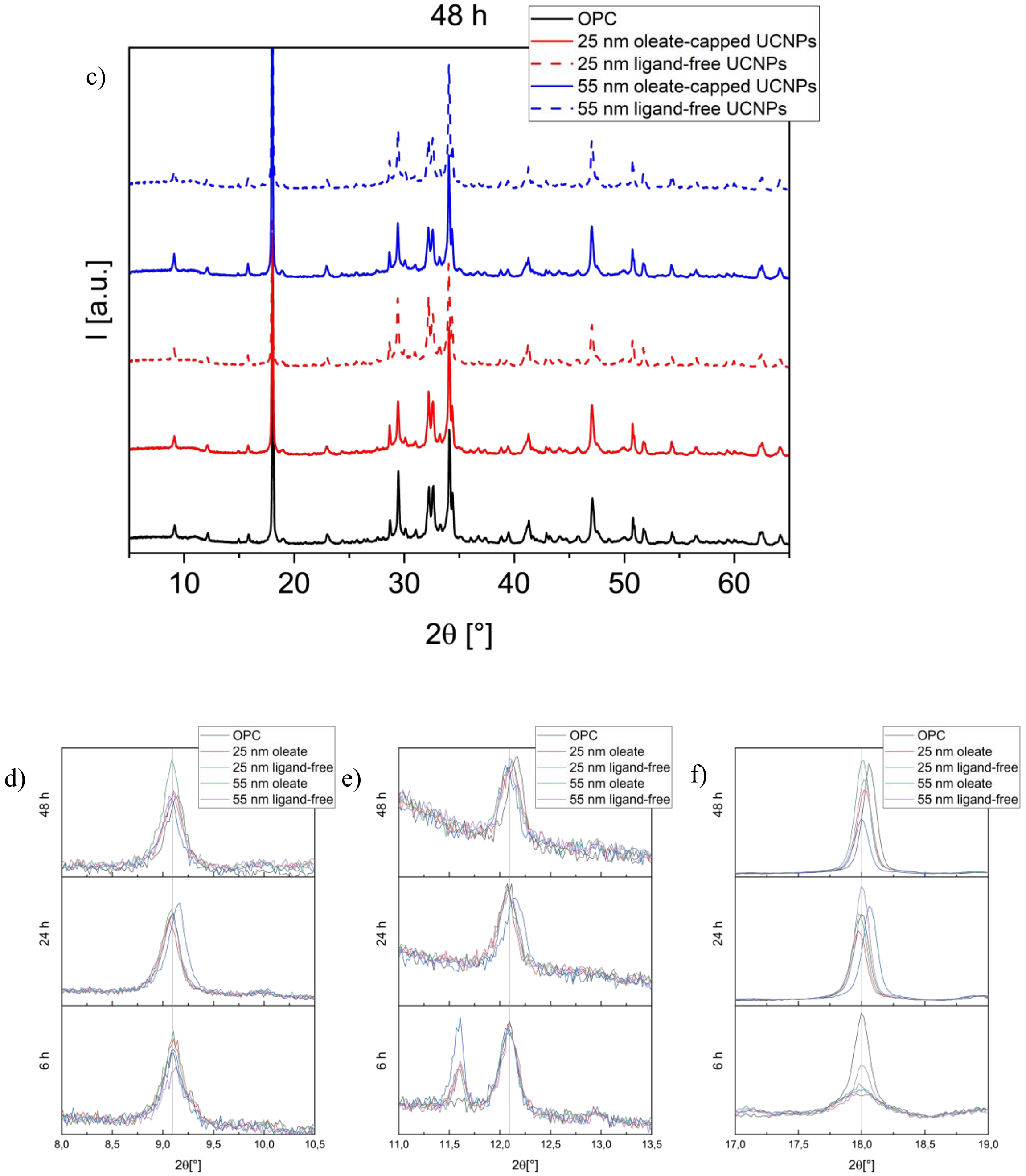

Figure SI 2: XRD-patterns of the UCNP-cement mixtures at a) 6 h, b) 24 h and c) 48 h of hydration. The patterns of the main reflexes at d) 9.1°, e) 12.1°, and f) 18° are also shown.

SI-3: Homogeneity of UCNP-cement samples

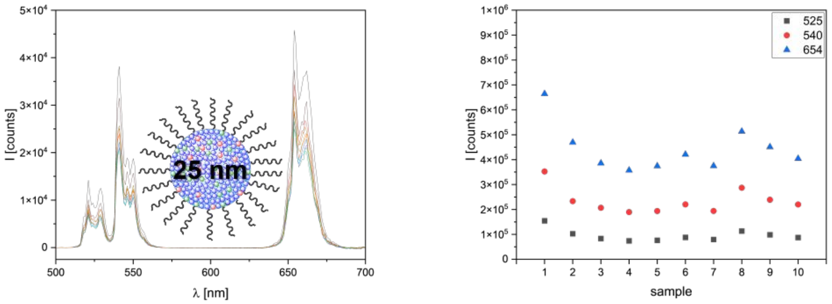

Figure SI 3: Emission measurements of 25 nm oleate-capped UCNPs at 1 wt% in OPC.

SI-4: Photoluminescence lifetime-measurements and fit-parameters:

Decay fits were done *via* Exponential component analysis (tail-fitting) with software FAST.

General Fit-formula: $Fit = \sum B_i \cdot e^{(\frac{-t}{\tau_i})}$ Formula for intensity average lifetime: $\tau_{int} = \frac{\sum_{i=1}^{n} B_i \tau_i^2}{\sum_{i=1}^{n} B_i \tau_i}$

Fit-Parameters:

25 nm oleate-capped UCNPs in OPC, hydration-time = 0 min, 540 nm band						
i	B_i	τ_i [ns]	Fraction [%]	χ^2	Decay background	τ_{int} [ns]
1	-2.00E+05	1.3e-8	5.7e-8	1.065	0.498	225,968
2	-49,335,352	100,648	13,997			
3	136,402,100	201,840	77.609			
4	6,630,617	449,063	8.394			

25 nm ligand-free UCNPs in OPC, hydration-time = 0 min, 540 nm band						
i	B_i	τ_i [ns]	Fraction [%]	χ^2	Decay background	τ_{int} [ns]
1	-47,094,824	98,426	13.137	1.005	0.519	224.282
2	-3,689,446	186,776	1.953			
3	134,104,883	199,820	75.943			
4	7,334,166	431,445	8.968			

25 nm oleate-capped UCNPs in OPC, hydration-time = 0 min, 654 nm band						
i	B_i	τ_i [ns]	Fraction [%]	χ^2	Decay background	τ_{int} [ns]
1	-2,540,109	80,366	0.690	1.022	0.824	199,514
2	-38,486,545	83,723	10.892			
3	131,656,328	174,630	77.718			
4	8,324,412	380,257	10.700			

25 nm oleate-capped UCNPs in OPC, hydration-time = 6 h, 654 nm band						
i	B_i	τ_i [ns]	Fraction [%]	χ^2	Decay background	τ_{int} [ns]
1	-7,925,447	38,631	1.958	1.066	1.501	179,612
2	19,689,650	114,002	14.357			
3	68,695,942	156,930	68.954			
4	6,585,350	349,732	14.731			

25 nm oleate-capped UCNPs in OPC, hydration-time = 12 h, 654 nm band						
i	B_i	τ_i [ns]	Fraction [%]	χ^2	Decay background	T_{int} [ns]
1	-10,623,937	45,463	2.232	1.070	0.649	172,014
2	117,884,736	133,547	72.743			
3	20,025,192	258,904	23.956			
4	274,616	842,444	1.069			

25 nm oleate-capped UCNPs in OPC, hydration-time = 24 h, 654 nm band						
i	B_i	τ_i [ns]	Fraction [%]	χ^2	Decay background	τ_{int} [ns]
1	-8,582,391	33,522	1.444	1.009	1.097	166,484
2	58,734,995	108,435	31.976			
3	68,583,027	166,240	57.242			
4	5,070,748	366,774	9.338			

25 nm ligand-free UCNPs in OPC, hydration-time = 0 h, 654 nm band						
i	B_i	τ_i [ns]	Fraction [%]	χ^2	Decay background	τ_{int} [ns]
1	2,206,291	19,187	0.434	0.866	0.488	194,010
2	-10,007,986	54,750	5.613			
3	47,560,459	171,161	83.392			
4	2,701,662	381,607	10.561			

25 nm ligand-free UCNPs in OPC, hydration-time = 6 h, 654 nm band						
i	B_i	τ_i [ns]	Fraction [%]	χ^2	Decay background	τ_{int} [ns]
1	-28,322,495	67,414	8.931	1.014	1.169	171,111
2	78,122,773	113,050	41.313			
3	48,889,375	186,907	42.745			
4	3,594,507	416,951	7.011			

25 nm ligand-free UCNPs in OPC, hydration-time = 12 h, 654 nm band						
i	B_i	τ_i [ns]	Fraction [%]	χ^2	Decay background	τ_{int} [ns]
1	-16,833,899	50,504	3.024	1.188	1.629	160,490
2	146,616,133	137,678	71.807			
3	24,178,376	268,641	23.106			
4	848,783	683,324	2.063			

25 nm ligand-free UCNPs in OPC, hydration-time = 24 h, 654 nm band						
i	B_i	τ_i [ns]	Fraction [%]	χ^2	Decay background	τ_{int} [ns]
1	45,293,530	0.158	0.053	1.004	0.960	176,063
2	67,042,588	148,308	73.491			
3	11,378,794	148,681	12.505			
4	5,442,750	346,807	13.952			

25 nm oleate-capped UCNPs in OPC, hydration-time = 1.5 a, 540 nm band						
i	B_i	τ_i [ns]	Fraction [%]	χ^2	Decay background	τ_{int} [ns]
1	267,251,504	58,414	57.901	1.092	3.640	149,665
2	64,795,083	133,592	32.105			
3	3,691,083	729,997	9.994			

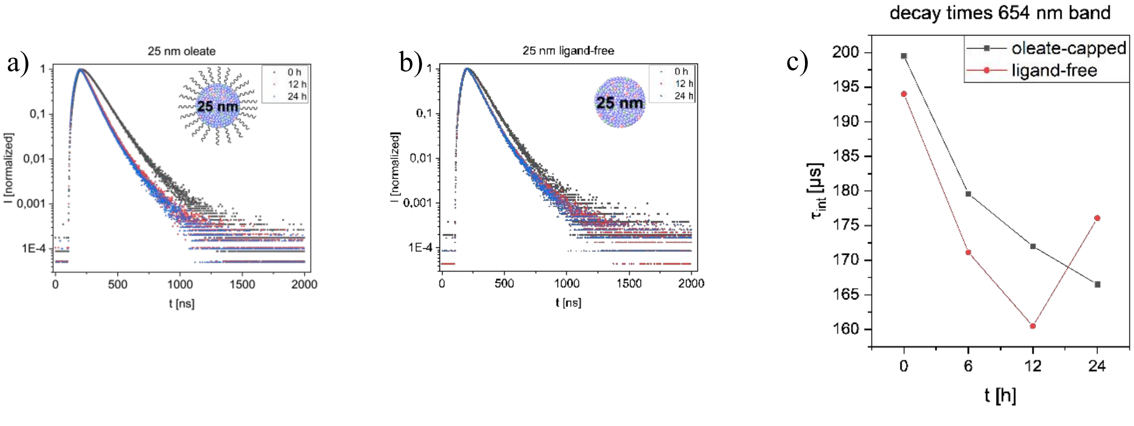

Figure SI 4: Fluorescence decay curves recorded at 540 nm of a) 25 nm oleate capped UCNPs, b) 25 nm ligand-free UCNPs in OPC at different times of cement hydration at w/c = 0.5 and c) graphical representation of the evolution of the intensity weighted lifetimes of he 654 nm emission band over 24 h of hydration.

SI-5: Emission spectrum of commercial upconversion particles in OPC

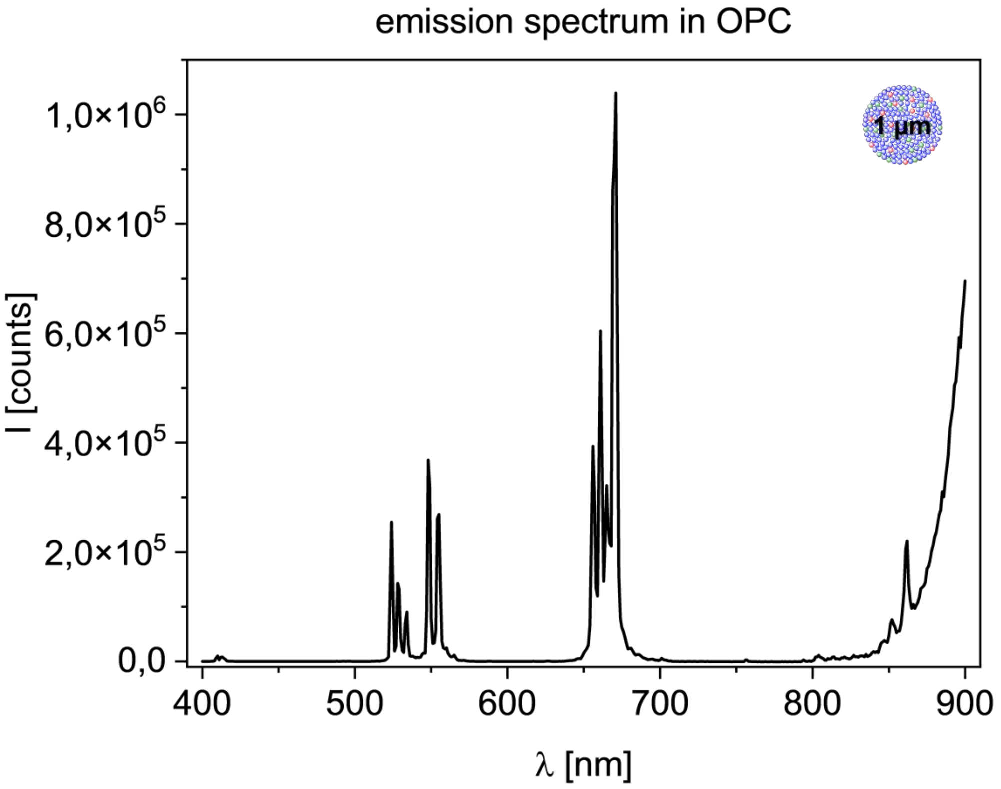

Figure SI 5: Emission spectrum of commercial upconversion particles in dry OPC; the loading concentration was at 1 wt%.
